# Physicochemical properties of superfine grinding-microwave modified artichoke soluble dietary fiber and their alleviation of alcoholic fatty liver in mice

**DOI:** 10.3389/fnut.2023.1253963

**Published:** 2023-08-16

**Authors:** Yayi Wang, Bian He, Linwei Zhang, Renwei Zhu, Liang Huang

**Affiliations:** ^1^School of Food Science and Engineering, Central South University of Forestry and Technology, Changsha, China; ^2^Hunan Key Laboratory of Processed Food for Special Medical Purpose, Changsha, China

**Keywords:** artichoke, soluble dietary fiber, compound modification, physicochemical properties, alcoholic fatty liver

## Abstract

The effects of superfine grinding (SG) and microwave treatment (MT) on the structure and physicochemical properties of artichoke soluble dietary fiber (ASDF) and its protective effects on mice with alcoholic fatty liver (AFL) were studied. We compared the changes in structural characteristics and physicochemical properties of ASDF, SG-ASDF (ASDF treated by SG), MT-ASDF (ASDF treated by MT), and CM-ASDF (ASDF treated by SG and MT). Moreover, we evaluated the effects of the obtained ASDF on the growth characteristics, blood lipid levels, and liver of mice with AFL. Our results of the study showed that CM-ASDF had a more concentrated and uniform particle size, a higher extraction rate of ASDF and significantly improved water-holding capacity (WHC), oil-holding capacity (OHC) and water swelling capacity (WSC) of ASDF (*p* < 0.05). After the ASDF intervention, mice with AFL exhibited a significant improvement in body lipid levels and reduce liver inflammation. Specifically, aspartate aminotransferase (AST), alanine aminotransferase (ALT), malonaldehyde (MDA), Tumor necrosis factor-α (TNF-α) and Interleukin-6 (IL-6) were significantly decreased, while superoxide dismutase (SOD) and glutathione peroxidase (GSH-PX) were significantly increased (*p* < 0.05). And the hematoxylin–eosin (HE) staining results showed significant improvement of hepatic steatosis in mice with AFL. In summary, our study found that both SG and MT could improve the structure and physicochemical properties of ASDF, with CM-ASDF being the most effective. Additionally, CM-ASDF was selected to continue the investigation and demonstrated an excellent protective effect on mice with AFL, with the high dose group (H-ASDF) showing the greatest benefit. These findings provided some new insights for future comprehensive utilization of ASDF and drug development for the treatment of AFL.

## 1. Introduction

Artichoke (*Cynara scolymus* L.) is a genus of artichokes in the family Asteraceae, native to the Mediterranean region and now cultivated worldwide (1). The main edible part of the artichoke is the bud surrounded by green leafy bracts (2), which is rich in functional components including polyphenols, sesquiterpenes, fatty acids, etc. (3). Consuming artichokes has been shown to have several health benefits, including protecting the kidney (4), reducing blood fat and blood sugar levels (5), improving gastrointestinal digestive function and preventing arteriosclerosis (6) and so on. However, with the increase in artichoke production, there is also an increase in waste, which is rich in dietary fiber (DF). Recycling valuable artichoke waste is an urgent challenge (7). Insoluble dietary fiber is the majority of ASDF, but its structure makes it not easily absorbed by human body (8). On the other hand, SDF not only significantly affects the metabolism of carbohydrates and lipids, but also adsorbs heavy metal ions and cholesterol, making it an important factor affecting the physiological function of DF (9, 10). Therefore, increasing the content of ASDF through modification has become a research hotspot.

There are several methods to modify DF, but physical methods have the advantages of technical simplicity, low cost, and sustainability compared to other methods (11). Superfine grinding (SG) is a novel physical modification method that can pulverize materials to 10–25 μm (12). This mechanical force disrupts the aggregation of intermolecular cellulose, hemicellulose and lignin to micronize DF (13). Hu et al. (14) found that green tea powder has more soluble carbohydrates after SG. Additionally, microwave-assisted extraction improves solubility by increasing the temperature and pressure inside the sample (15). Gan et al. (16) modified the SDF of grapefruit peel using three different microwave-assisted modifications and found significant improvements in functional properties such as WHC and OHC. Although there are currently some data on the impact of SG and MT combined with other modification methods on DF, there has been almost no exploration about the effect of the composite modification of SG and MT on DF.

Alcoholic fatty liver (AFL) is a fatty disease of the liver caused by long-term excessive consumption of alcohol (17). Large amounts of alcohol entering the body can cause liver damage and lead to fat accumulation in the liver and steatosis (18). If left untreated, AFL can progress to steatohepatitis, liver fibrosis, cirrhosis or even cancer (19). The incidence rate and mortality of AFL are increasing worldwide. While restricting alcohol intake is an effective treatment, related lipid-lowering or anti-inflammatory drugs have limited efficacy. Therefore, it is urgent to develop more effective and practical drugs. Tsukada et al. (20) found that when alcohol intake was 36% of total calories, combined intake with high dietary carbohydrate could inhibit the deterioration of AFL in mice by increasing hepatic cytochrome CYP2E1 activity.

In this paper, the effects of microwave and superfine grinding on the structure and physicochemical properties of ASDF were investigated. Besides, we also probed into the protective effect of ASDF on AFL mice to provide a theoretical basis for the development of ASDF and the effective reuse of artichoke waste fraction.

## 2. Materials and methods

### 2.1. Materials and reagents

Artichoke was obtained from Huimei Agricultural Technology Co., Ltd. All other chemicals were of analytical grade. Basic feed (35.6% protein, 44.3% fat, 20.1% carbohydrate) and high fat feed (88% basic feed, 1.5% cholesterol, 5% lard, 0.5% sodium cholate, 5% egg yolk powder) were provided by Beijing Keao Xieli Feed Co., Ltd. The kits used for measuring of GSH-PX, SOD, MDA were obtained from Nanjing Jiancheng Bioengineering Institute. The kits used for measuring AST and ALT were provided by Shenzhen Mindray Bio-medical Electronics Co. Ltd. Hongxing Erpotou Wine 56%(v/v) liquor, a 56%(v/v) aqueous ethanol solution, was purchased from Beijing Hongxing Co., Ltd.

### 2.2. Sample preparation

ASDF was obtained by adding 1% citric acid solution to the crushed artichoke powder at a material-liquid ratio of 1:25 (w/v), soaking for 30 min and then extracting the mixture. The filtrate was retained, and the precipitate was obtained by adding an equal amount of citric acid solution for a second extraction. Finally, the two filtrates were combined to obtain ASDF. The filtrate was concentrated by evaporating (75°C). Next, four times the volume of anhydrous ethanol was added to the concentrate, and let it precipitated for 12 h. The sediment was collected and dried to constant weight at 60°C to obtain ASDF, which was used for further study. SG-ASDF was obtained by extraction after ASDF superfine grinding (material ratio, ASDF: zirconium ball = 1:4 (w/w); crushing time, 8 h). MT-ASDF was obtained by microwave treatment (microwave time, 30 s; microwave power, 500 W).CM-ASDF (material ratio, ASDF: zirconium ball = 1:4 (w/w); crushing time, 8 h) was obtained after composite modification with SG and MT. The purities of ASDF, SG-ASDF, MT-ASDF and CM-ASDF were 65.59 ± 0.46 g/100 g, 70.89 ± 0.10 g/100 g, 68.50 ± 0.19 g/100 g and 71.72 ± 0.58 g/100 g, respectively.

### 2.3. Determination of particle size

The particle size was determined by laser particle size analyzer JL-1178 (Chengdu Jingxin Powder Instrument Co., Ltd., Chengdu, China), and air was used as the dispersant. The sample was dispersed uniformly with the aid of ultrasonic waves. The refractive index and medium refractive index were 1.510 and 1.33, and particle size measurement was from 0.01 μm to 2000 μm. The width of the particle size distribution was calculated by Equation (1):


(1) $$span=\frac{\left(D_{90}-D_{10}\right)}{D_{50}}$$

where D10 is the size of the sample with a cumulative size distribution of 10%, D50 is the size of the sample with a cumulative size distribution of 50% and D90 is the size of the sample with a cumulative size distribution of 90%.

### 2.4. Scanning Electron Microscopy (SEM)

The sample structure was measured by an FEG-250 scanning electron microscope (FEI Company, Hillsboro, OR, USA). An appropriate amount of dry sample was fixed on the sample seat and the height of the sample was adjusted. The accelerating voltage was 5 kV and the working distance was 10 mm. The morphology of the sample particles was observed at 500 × and 5,000 × magnification conditions, respectively. The photographs of different magnifications were taken to analyze the changes in the structural morphology of the samples.

### 2.5. Fourier Transform Infrared Spectroscopy (FTIR)

The FTIR analysis was performed with FTIRTracer-100 (SHIMADZU Corporation, Kyoto, Japan) from 400 to 4,000 cm^−1^ with 32 scans, and the scanning resolution was 4 cm^−1^. 2 mg of the dried sample was mixed with 200 mg of dried KBr powder by complete fine-grinding into a pressed film, which was pressed into a piece and scanned with KBr as the blank background.

### 2.6. Physicochemical properties analysis

#### 2.6.1. Water-Holding Capacity (WHC)

The method was conducted in triplicate according to that described by Li et al. (21) with slight modifications. 8 mL of pure water was mixed with 0.1 g of sample and shaken at 25°C for 2 h. After centrifugation at 4000 × rpm for 10 min, the precipitate was collected and weighed. WHC was calculated by Equation (2):


(2) $$\mathrm{WHC}\left(g/g\right)=\frac{W-W_{1}}{W_{1}}$$

where W is the wet weight, and W_1_ is the dry weight.

#### 2.6.2. Oil-Holding Capacity (OHC)

The method was conducted in triplicate according to a previous reported (22) with slight modifications. The 0.5 g sample was mixed with 10 mL peanut oil for 30 min and then centrifuged at 4000 r/min for 10 min, then the excess oil was discarded and the weight of the remaining precipitate was weighed. OHC was calculated by Equation (3):


(3) $$\mathrm{OHC}\left(g/g\right)=\frac{W-W_{1}}{W_{1}}$$

where W is the wet weight, and W_1_ is the dry weight.

#### 2.6.3. Water Swelling Capacity (WSC)

The method was conducted in triplicate as previously described by Zhang et al. (23) with slight modifications. 10 mL of pure water was mixed thoroughly with 1 g of sample in a centrifuge tube and left at 25°C for 24 h. WSC was calculated by Equation (4):


(4) $$\mathrm{WSC}\left(\mathrm{ml}/g\right)=\frac{V_{1}-V_{2}}{W_{1}}$$

where V_1_ is the volume after expansion; V_2_ is the volume of the dry sample; W_1_ is the dry weight of the sample.

### 2.7. XRD

The sample was collected for X-ray diffractograms with an X-ray diffractometer smarlab ragiku 2019 (SHIMADZU Corporation, Kyoto, Japan) at 1500 mA and 40 kV current and operating voltage. The diffraction angle (2θ) was scanned in the range of 4° to 90° with a scanning speed of 2°/min. The crystallinity index (IC) was calculated according to the Scherrer method.

### 2.8. Animals and experimental method

Fifty SPF-grade C57BL/6j male mice, free of specific pathogens and weighing approximately 23 ± 3 g/each, were provided by Hunan SJA Laboratory Animal Co., Ltd. (animal production license number: SCXK (Xiang) 2016–0002 (Changsha, China)). Before the start of the experiment, mice were acclimatized at 24 ± 2°C and 12 h of light for 1 week. All experimental procedures and facilities were approved by the Animal Ethics Committee of Hunan SJA Laboratory Animal Co., Ltd. with project identification code IACUC-SJA2021020 and approval date of November 14, 2021. The mice were randomly divided into 5 groups of 10 mice each: blank group (BG), control group (MG), low-dose group (L-ASDF), medium-dose group (M-ASDF) and high-dose group (H-ASDF). The mice were housed continuously for 4 weeks according to the above groupings. During the experiment, the state of the mice was observed every day, including appetite, mental state, etc. The blank group was fed with basic chow and saline solution, the remaining 4 groups were fed high-fat chow and Hongxing Erhuotou ethanol aqueous solution. The L-ASDF, M-ASDF and H-ASDF were provided with CM-ASDF and saline prepared to a concentration of 0.4, 0.8, and 1.6 g/mL, respectively, every day, respectively, twice a week with white spirit. After the 4-week period fasting and water fasting for 12 h, all mice were anesthetized with isoflurane respiration at a dose of 100 mg/kg and rapidly deprogrammed. The blood was taken from the eyes, and the livers were dissected and weighed. Some livers were fixed in paraformaldehyde and stored on dry ice for the determination of liver histopathological sections and other indexes. The remaining livers were stored in the refrigerator at −80°C for further analysis and testing.

### 2.9. Determination of serum lipids and AST and ALT activities in mice

Blood samples of mice was taken from their eyes and the serum was collected after centrifugation at 3000 r/min for 20 min. The activities of AST and ALT were measured with the corresponding kits. TC, TG, LDL-C and HDL-C were measured with a FAITH-1000 fully automated biochemical analyzer (Nanjing Laura Electronics Co., Ltd., Nanjing, China).

### 2.10. Determination of antioxidant index and inflammatory cytokine levels in serum of mice

A certain amount of liver tissue was taken with saline to make a homogenate, which was centrifuged at 3000 r/min for 15 min. The SOD and GSH-PX enzyme activities, as well as the MDA content were measured with the corresponding kits.

The liver tissue of mice and the pre-cooled normal saline were homogenized, and the supernatant was centrifuged at 10000 r/min for 10 min and then placed at 4°C to be measured. The levels of IL-6 and TNF-a were measured with commercial ELISA kits.

### 2.11. Histopathological analysis of mice liver

The mice liver tissues were fixed with 4% paraformaldehyde for 48 h, dehydrated with alcohol, embedded in xylene, sectioned, and stained with Hematoxylin–eosin (HE). The stained sections were then observed under a light microscope.

### 2.12. Statistical analyses

All experiments were performed in triplicates except for the mice in the animal experiments. Data were analyzed by SPSS 22.0 statistical software, and the results were expressed as mean ± standard deviation (S.D.). The paired t-test and one-way ANOVA were used to determine the significance of differences.

## 3. Results and discussion

### 3.1. Determination of ASDF particle size

The particle size distributions of ASDF, MT-ASDF, SG-ASDF and CM-ADF are displayed in Table 1. The results revealed that microwave, SG, and CM treatment reduced the particle size of ASDF. The volume-area-averaged particle size of CM-ASDF was the smallest. Noteworthy, the particle size distribution of ASDF was concentrated, but after treatment, the particle size of CM-ASDF was more concentrated and uniform than that of SG-ASDF and MT-ASDF.

**Table 1:** Particle size distribution of ADF, MT-ADF, SG-ADF and CM-ADF.

Group	D10 (μm)	D50 (μm)	D90 (μm)	Span	D [3,2] (μm)
ASDF	234.05 ± 0.82^a^	493.62 ± 5.10^a^	786.93 ± 2.31^a^	1.12 ± 0.01^a^	4.10 ± 0.04^a^
MT-ASDF	14.26 ± 0.51^b^	146.82 ± 3.15^b^	409.61 ± 6.93^b^	2.69 ± 0.08^b^	0.31 ± 0.02^b^
SG-ASDF	3.21 ± 0.12^c^	92.09 ± 1.08^c^	282.06 ± 3.26^c^	3.03 ± 0.11^c^	0.21 ± 0.02^c^
CM-ASDF	2.42 ± 0.05^d^	83.89 ± 0.93^d^	176.93 ± 1.78^d^	2.08 ± 0.02^d^	0.10 ± 0.01^c^

D [3,2] is the volume-area-averaged particle size.

Data are expressed by mean ± standard deviation. Values with different letters in the same column are significantly different (*p* < 0.05).

During the superfine grinding, ASDF and zirconium balls were fully rubbed and collided during the crushing process, and the degree of crushing was better than the traditional crushing method. After microwave, the ambient temperature and pressure increase significantly, and its particles were further broken, so that the particle size was further reduced and more uniformly distributed (12). Compared with SG-ASDF, MT-ASDF had a more concentrated particle size distribution, but the latter had an overall smaller particle size. A further reduction of the volume-area-averaged particle size was clearly observed when ASDF was treated with a combination of MT and SG. Gong (24) reported that the cell wall of CM-ASDF from peanut shell was much more damaged than by other treatment methods. This might be due to the fact that after the combination of MT and SG, its internal structure was fully opened.

### 3.2. Scanning Electron Microscopy (SEM)

**Figure 1:**
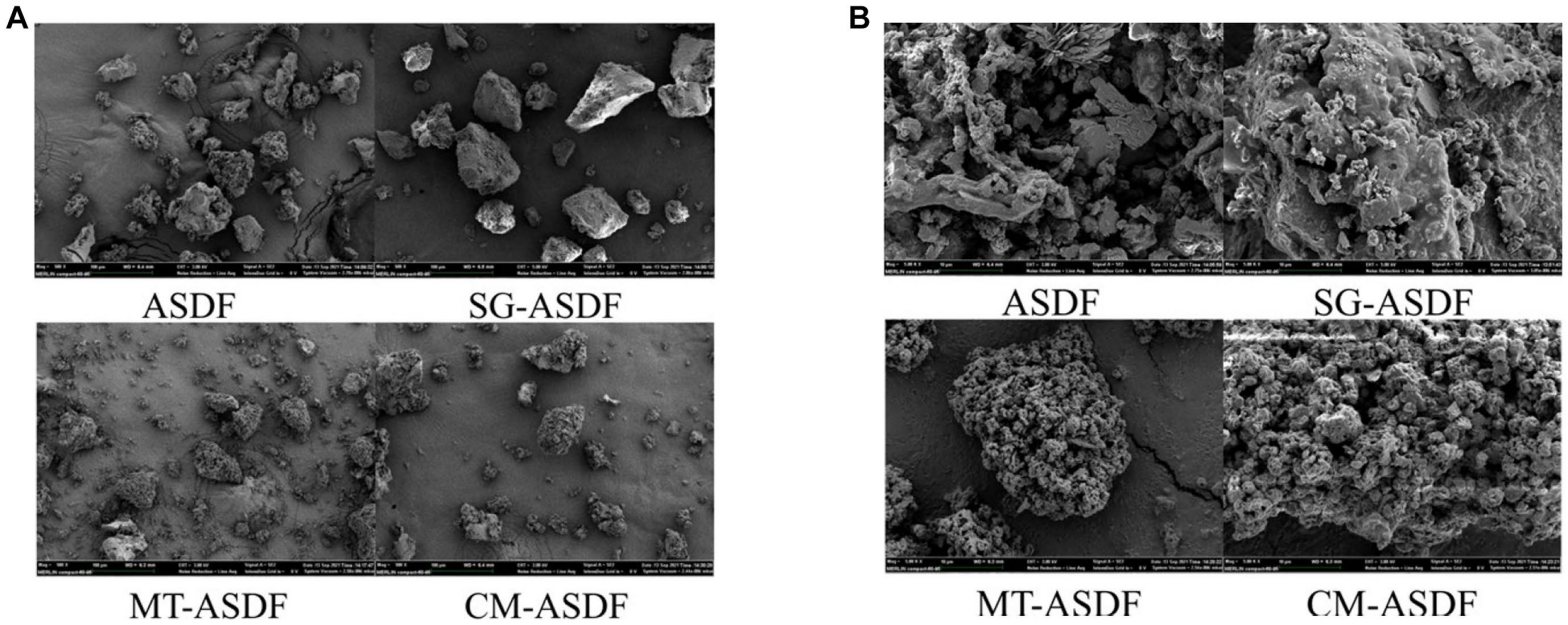
Scanning electron micrograph of ASDF. **(A)** 500 × magnification; **(B)** 5,000 × magnification.

The SEM images of ASDF subjected to different treatment methods were displayed in Figure 1. The particle sizes of SG-ASDF and CM-ASDF were all lesser than ASDF and MT-ASDF (Figure 1A). ASDF was processed into finer particles by superfine grinding. This was consistent with the results reported by Zhu et al. (25). But compared to SG-ASDF, to the combined treatment promoted a better degree of ASDF particle pulverization. In addition, the structure of ASDF was damaged to a certain extent after superfine grinding, thus the hydrophilic and lipophilic groups are exposed. The combined effect of SG and MT increased the degree of crushing of ASDF particles which formed a much looser porous fiber structure and increased specific surface area, so that the physicochemical properties of ASDF (26) (Figure 1B).

### 3.3. FTIR spectra

**Figure 2:**
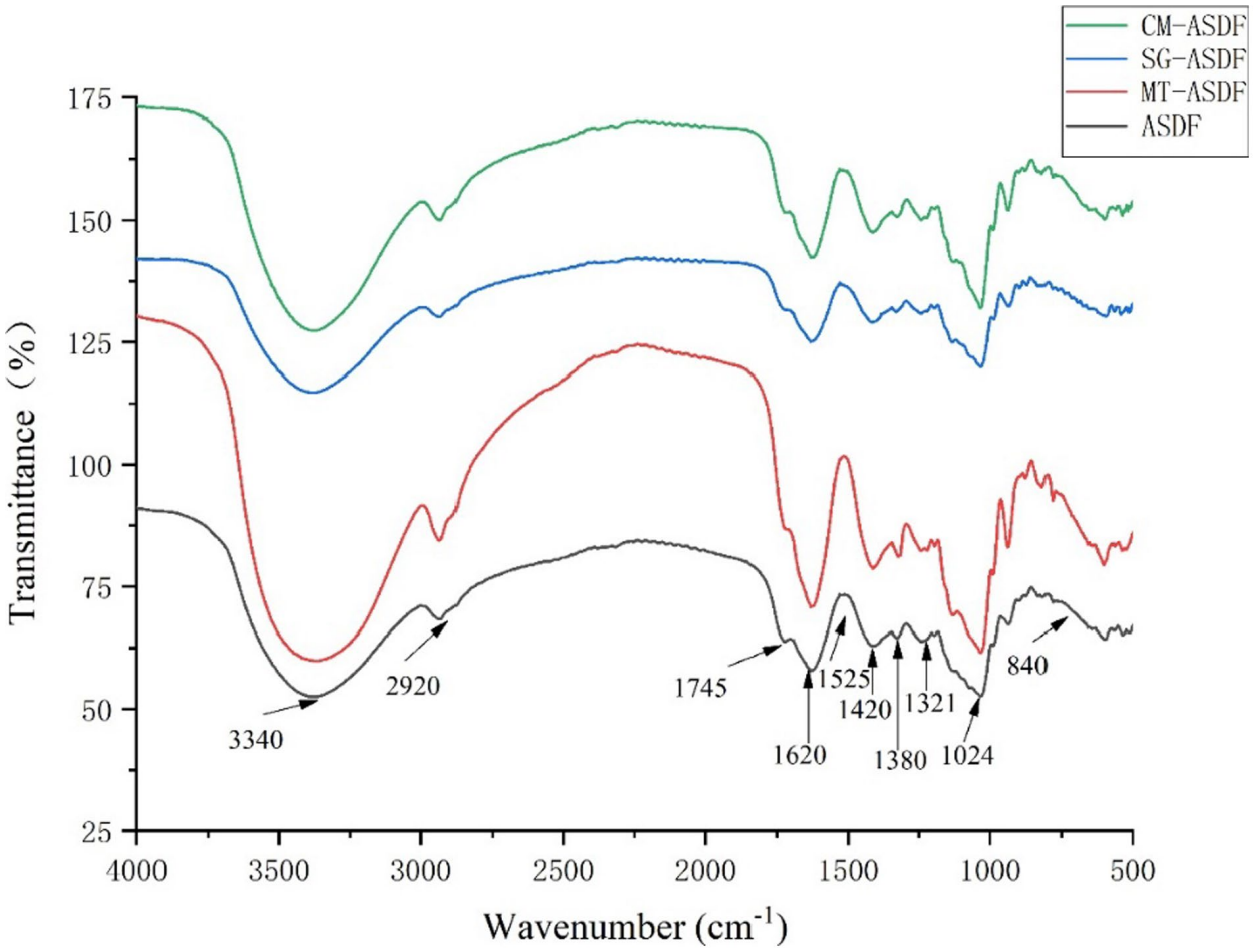
FTIR spectra of the ASDF, MT-ASDF, SG-ASDF and CM-ASDF.

From Figure 2, it can be seen that there was a strong signal at 3340 cm^−1^, which indicated that the hydroxyl groups of cellulose and hemicellulose provide hydrogen bonding to vibrate (23), and CM-ASDF had the strongest vibration and an increase in peak intensity. The absorption peak at 2920–2925 cm^−1^ was attributed to the polysaccharide methyl or methylene C-H stretching band (27). The peak near 1745 cm^−1^ was attributed to the hemicellulose (28). The absorption peak at 1620–1630 cm^−1^ was the H-O-H bending vibration peak of adsorbed water, and the peak at 1417–1420 cm^−1^ was the CH_2_ bending vibration peak in cellulose (29). The peak near 1,380 cm^−1^ was attributed to the aliphatic asymmetric C-H bond bending vibration peak, and the peak at 1321–1324 cm^−1^ was attributed to the bending vibration peak of CH_2_ in carbohydrates, and the peak at 1017–1024 cm^−1^ was the C-O stretching vibration peak in carbohydrates. The absence of the characteristic absorption peak of lignin at 1530 cm^−1^ indicated that all four sets of samples do not contain lignin (30). The results showed that ASDF had special absorption peaks such as C-H bond, C=O bond, H-O-H bond, CH_2_ bond, and C-O bond, and no new absorption peaks appeared after the modification. Therefore, the chemical composition of ASDF was not affected by the four treatments. The alkyl and oxygen-containing functional group peak intensities gradually increased for CM-ASDF compared to the other three treatments, which might indicate that the composite modification method treated ASDF was extracted at a higher rate.

### 3.4. WHC, OHC, and WSC

The WHC, OHC and WSC of ASDF, MT-ASDF, SG-ASDF and CM-ASDF were shown in Table 2. The different treatments resulted in a significant increase in WHC, OHC and WSC of ASDF, which was consistent with the findings of a previous report (26). The analysis revealed that ASDF treated with SG and microwave exposed more hydrophilic groups, which improved the WHC of ASDF. Gan et al. (16) found that microwave-assisted modification resulted in a significant increase in OHC of SDF in grapefruit peels. The OHC of CM-ASDF was significantly higher than the other groups (*p* < 0.05). The surface of CM-ASDF was more porous so that the specific surface area was larger than the other groups, which facilitated the adsorption of oil and grease, leading to higher the OHC. This property was beneficial to the retention of oil and grease in food during processing. WSC was an important indicator of SDF quality, and a higher value usually indicated better adsorption performance (31). The hydrophilic groups exposed by CM-ASDF have an affinity for water molecules, causing them to swell to create a bulk effect. This improves satiety, decrease food intake, reducing obesity and promote overall human health (32).

**Table 2:** Effect of MT, SG, CM on the WHC, OHC and WSC of ASDF.

Group	WHC (g/g)	OHC (g/g)	WSC (ml/g)
ASDF	4.86 ± 0.02^d^	2.35 ± 0.02^d^	4.45 ± 0.02^d^
MT-ASDF	5.63 ± 0.02^c^	4.20 ± 0.02^c^	5.32 ± 0.03^c^
SG-ASDF	6.21 ± 0.02^b^	4.87 ± 0.02^b^	6.14 ± 0.02^b^
CM-ASDF	7.83 ± 0.01^a^	5.05 ± 0.03^a^	7.07 ± 0.15^a^

Data are expressed by mean ± standard deviation (*n* = 3). Values with different letters in the same column are significantly different (*p* < 0.05).

### 3.5. XRD

**Figure 3:**
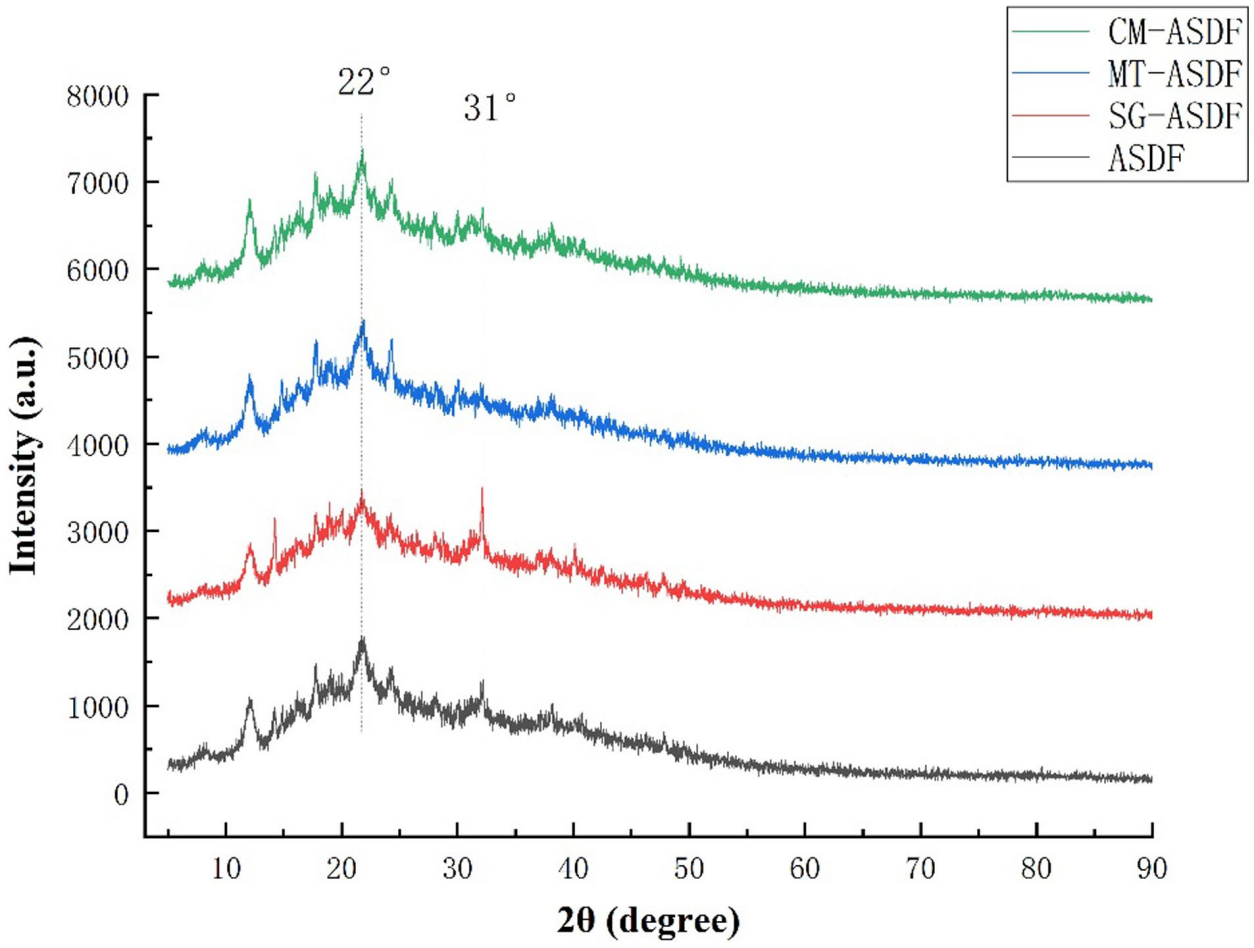
XRD spectra of the ASDF, MT-ASDF, SG-ASDF and CM-ASDF.

DF extracted from plants usually consists crystalline regions due to the presence of cellulose and amorphous regions which are formed by amorphous cellulose, hemicellulose, and lignin (33). XRD was used to analyze the effect of different modification methods on the crystal structure of SDF. The X-ray diffraction patterns of ASDF, MT-ASDF, SG-ASDF, and CM-ASDF were shown in Figure 3. ASDF had a strong diffraction peak at 22° (2θ) and a weaker diffraction peak at 31°(2θ), which was characteristic of the cellulose crystal structure (34). It indicated that ASDF was a state where crystalline and amorphous regions co-exist (35). Compared with other methods, the position of diffraction peaks of CM-ASDF did not change significantly, which proved that the crystalline configuration of ASDF did not change significantly, indicating that the composite modification method did not cause significant damage to the crystalline region of ASDF.

### 3.6. Effects of ASDF on serum TC, TG, LDL-C, HDL-C, AST and ALT levels in mice

The serum TC and TG, as well as LDL-C and HDL-C levels, in mice are presented in Table 3. Compared with the BG group, the MG group exhibited significant increases in TC, TG, and LDL-C levels by 83.69, 136.36, and 224.14%, respectively, while HDL-C significantly decreased by 41.75% (*p* < 0.05). These results indicated that the liver fat metabolism of mice was abnormal after continuous intake of high-fat diet and alcohol. Throughout the feeding period, the levels of TC, TG, and LDL-C in mice in the L-ASDF, M-ASDF, and H-ASDF groups tended to decrease compared with those in the MG group, with the most significant decreases observed in the H-ASDF group by 15.52, 55.38, and 25.53%, respectively. Moreover, when compared with the MG group, the HDL-C levels in the L-ASDF, M-ASDF and H-ASDF groups showed a more pronounced increase, with the most obvious one in the H-ASDF group, which increased by 68.07%. The results indicated that ASDF can effectively improve the blood lipid level in mice, with higher doses showing a more significant effect. With increasing intake, ASDF could effectively improve serum lipid accumulation in mice. The exposed hydrophilic and lipophilic groups of ASDF after superfine grinding and microwave treatment elevated the WHC, OHC, and WSC of ASDF, which better promoted adsorption of cholesterol-fat as well as the metabolism of alcohol and fat by livers, and thus exerted a mitigating effect on AFL.

**Table 3:** Serum lipids in mice.

Group	TC (mmol/L)	TG (mmol/L)	LDL-C (mmol/L)	HDL-C (mmol/L)
BG	2.33 ± 0.06	0.55 ± 0.09	0.29 ± 0.04	2.85 ± 0.09
MG	4.28 ± 0.12	1.30 ± 0.04^*^	0.94 ± 0.63^*^	1.66 ± 0.11^*^
L-ASDF	4.20 ± 0.09^*^	1.06 ± 0.07^*△^	0.81 ± 0.08^*^	2.30 ± 0.13^*△^
M-ASDF	4.09 ± 0.24^*^	0.69 ± 0.05^△^	0.77 ± 0.07^*^	2.68 ± 0.09^△^
H-ASDF	3.53 ± 0.31^*△^	0.58 ± 0.08^△^	0.70 ± 0.05^*△^	2.79 ± 0.07^△^

Data are reported as mean ± standard deviation from ten Mice in each group. *p < 0.05 compared with the blank group (BG), ^△^*p* < 0.05 compared with the model group (MG).

Long-term alcohol intake could cause abnormal accumulation and degeneration of fat in the liver, which could lead to liver damage (36). The levels of serum AST and ALT in mice are shown in Table 4. AST and ALT are mainly found in various cells, with the highest concentration in liver cells. In cases of the liver damage or hepatocytes destruction, large amounts of AST and ALT in hepatocytes are released into the bloodstream, leading to a sharp increase in AST and ALT. This elevation in AST and ALT serves as crucial diagnostic indicators for AFL (37). In the MG group, both AST and ALT levels were significantly higher, representing a 41.67 and 64.15% increase compared with the BG group (*p* < 0.05), and both aminotransferase levels were significantly elevated (*p* < 0.05). This indicated that during feeding, alcohol caused more serious damage to the liver cell membranes of mice, resulting in an increase in their cell membrane permeability and the release of large amounts of AST and ALT into the blood. Compared with the MG group, the L-ASDF, M-ASDF, and H-ASDF groups exhibited reductions in AST and ALT levels by 1.23, 9.77, 15.32 and 7.73%, 18.37, 30.17% respectively, with the most significant decrease in the H-ASDF group. These results indicate that ASDF has a certain protective effect on liver cells and kidneys by reducing AST and ALT in serum, thereby reducing liver damage caused by alcohol intake.

**Table 4:** Serum AST and ALT levels in mice.

Group	AST (U/ml)	ALT (U/ml)
BG	31.80 ± 1.40	105.89 ± 2.05
MG	52.20 ± 1.56^*^	150.01 ± 3.69^*^
L-ASDF	47.25 ± 0.96^*^	149.38 ± 4.18^*^
M-ASDF	43.18 ± 0.76^*^	141.82 ± 3.82^*^
H-ASDF	41.42 ± 1.03^*△^	123.88 ± 9.43^*△^

Data are reported as mean ± standard deviation from ten Mice in each group. *p < 0.05 compared with the blank group (BG), ^△^*p* < 0.05 compared with the model group (MG).

### 3.7. Effects of ASDF on serum antioxidant index and inflammatory cytokine levels in mice

As shown in Table 5. The SOD of mice in MG group was 65.85% lower than that in BG group, indicating the more severe damage of alcohol on the liver during feeding. The liver had a strong antioxidant defense in normal physiological circumstance, and after the intake of alcohol, the body’s antioxidant capacity was reduced (38). SOD is an essential antioxidant enzyme that plays a vital role in the balance of oxidation and antioxidation in the body (37). SOD in H-ASDF group was significantly increased by 97.32% compared with MG group (*p* < 0.05). MDA can reflect the severity of lipid peroxidation and liver injury in liver tissue (39). The content of MDA in liver of mice in MG group was relatively high, reaching 6.24 mmol/L, respectively, and was significantly decreased by 79.48% in H-ASDF group (p < 0.05). GSH-PX enhances the body’s ability to resist oxidative damage by scavenging free radicals. The activity level of GSH-PX in the MG group was 27.59% higher than that in the BG group, and compared with MG group, the activity of GSH-PX in the liver of L-ASDF group, M-ASDF group and H-ASDF group was increased by 7.48, 13.35 and 26.42%. In conclusion. ASDF had the ability of scavenging free radicals, which might be due to the combination of free radicals exposed to ASDF after SG and MT with free radicals in mice, thereby inhibiting lipid peroxidation and alleviating oxidative stress in mice liver caused by alcohol intake.

**Table 5:** Effect of ASDF on SOD, MDA and GSH-PX activity in mice liver.

Group	SOD (U/ml)	GSH-PX (U/mg)	MDA (mmol/l)
BG	19.65 ± 0.64	66.29 ± 2.80	1.04 ± 0.19
MG	6.71 ± 0.39^*^	48.00 ± 2.69^*^	6.24 ± 0.24^*^
L-ASDF	10.33 ± 0.54^*△^	51.59 ± 0.76^△^	4.07 ± 0.30^*△^
M-ASDF	11.33 ± 0.77^*△^	54.41 ± 0.91^△^	3.05 ± 0.37^*△^
H-ASDF	13.24 ± 0.87^*△^	60.68 ± 0.98^*△^	1.28 ± 0.16^△^

Data are reported as mean ± standard deviation from ten Mice in each group. *p < 0.05 compared with the blank group (BG), ^△^*p* < 0.05 compared with the model group (MG).

TNF-α plays an important role in apoptosis, cell survival, inflammation and immunity. Appropriate amount of TNF-α can regulate the immune function of the body, maintain the normal and stable state of the body, and resist various pathogenic factors (40). IL-6 is a major cytokine in chronic subclinical inflammation and regulates liver inflammation and regeneration (41). As shown in Table 6, it is evident that both TNF-α and IL-6 were significantly higher in the MG group, representing a 36.69 and 46.22% increase compared with the BG group (*p* < 0.05). After liver injury, TNF-α increases dramatically, TNF-α binds to receptors to cause apoptosis and stimulates signaling pathways to secrete inflammatory factors such as IL-6, further aggravating liver cell lesions and degeneration (42). Compared with the MG group, TNF-α and IL-6 levels decreased in all three different dose groups, with serum IL-6 levels returning to normal in the H-ASDF group (p < 0.05). It indicates that the intake of ASDF reduced hepatic lipid accumulation and decreased the degree of hepatic steatosis and hepatic inflammatory cytokine levels. In conclusion, ASDF can attenuate liver inflammation in alcoholic fatty liver mice by inhibiting TNF-α pathway and IL-6 secretion.

**Table 6:** Effect of ASDF on inflammatory cytokine levels in mice liver.

Group	IL-6 (pg/mg prot)	TNF-α (pg/mg prot)
BG	209.01 ± 65.04	161.19 ± 54.33
MG	285.69 ± 33.40^*^	235.69 ± 59.38^*^
L-ASDF	238.33 ± 56.91^*△^	201.93 ± 26.45^*△^
M-ASDF	230.36 ± 30.93^△^	196.84 ± 33.18^*△^
H-ASDF	220.27 ± 12.93^△^	179.58 ± 26.52^*△^

Data are reported as mean ± standard deviation from ten Mice in each group. *p < 0.05 compared with the blank group (BG), ^△^*p* < 0.05 compared with the control group (MG).

### 3.8. Protective effect of ASDF on liver tissue in mice

**Figure 4:**
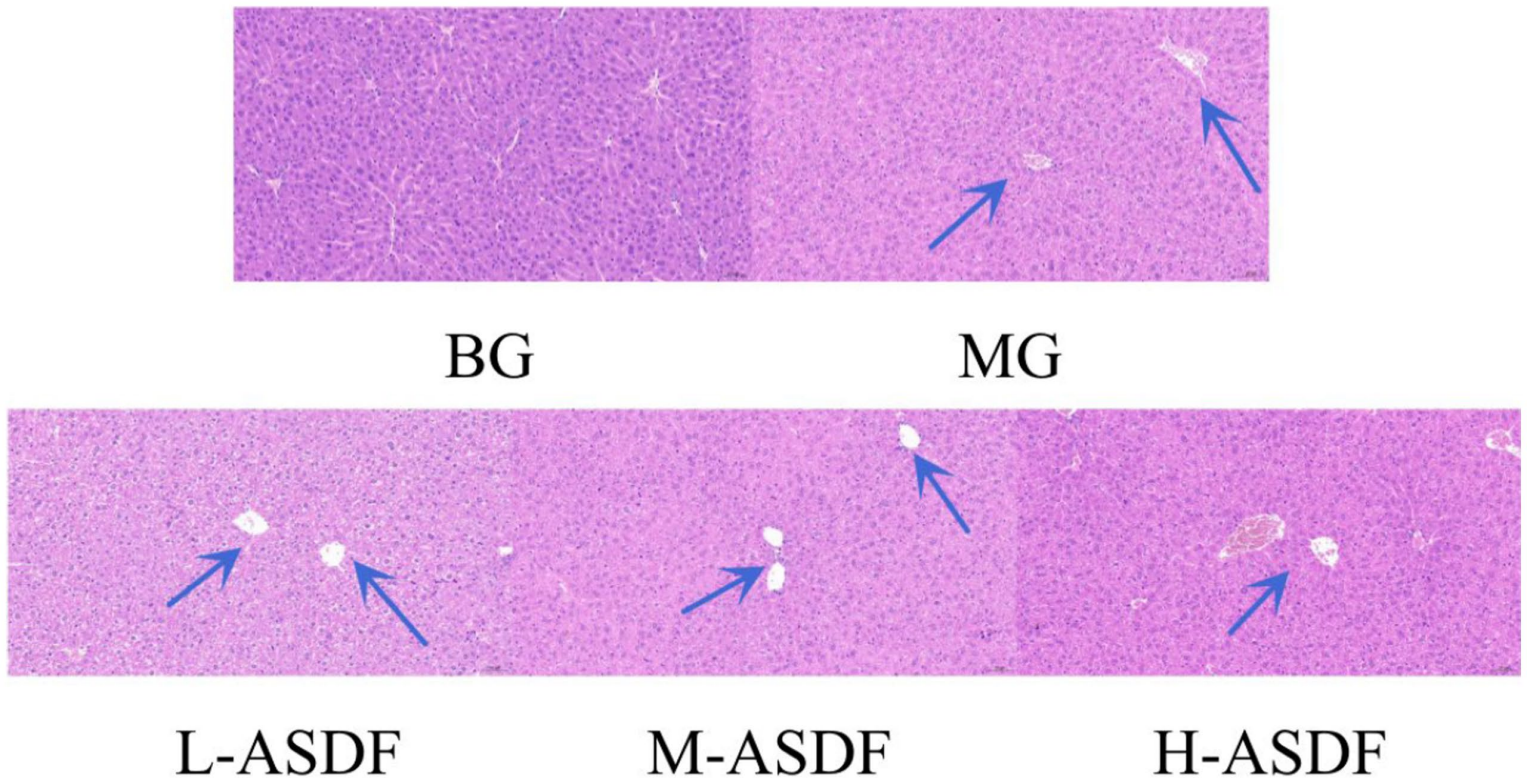
Hematoxylin–eosin-stained section of mice liver (20 × magnification). From left to right: blank group (BG) with normal appearance, control group (CG), L-ASDF group, M-ASDF group and H-ASDF group. Round vacuoles indicated by the blue arrow indicate hepatocyte steatosis.

As shown in Figure 4, BG group exhibited normal liver tissue structure, with uniform cell coloration and typical liver tissue morphology. In contrast, the MG group displayed long fat cavities, swollen and deformed hepatocytes, and significant infiltration of inflammatory cells. Comparatively, the L-ASDF, M-ASDF, and H-ASDF groups showed a significant reduction in fat vacuoles and alleviated hepatocyte swelling compared to the MG group. However, it is important to note that although there was improvement, the amount of fat vacuoles in these groups did not reach the level observed in healthy liver tissue. This suggests that while ASDF can provide a certain degree of protection, it cannot fully replace pharmaceutical interventions for treatment purposes.

A large amount of hepatocellular steatosis was visible in the MG group (38), and white round vacuoles of large size were clearly seen (blue arrows). ASDF intervention improved intracellular steatosis in the liver of mice in the three different dose groups, with the H-ASDF group being the most pronounced.

## 4. Conclusion

By comparing the changes in the structural characteristics and physicochemical properties of ASDF treated by SG, MT and the combination of the two methods, we found that the physicochemical properties of CM-ASDF were better and the particle size was more uniform and concentrated. Feeding high doses of CM-ASDF to mice with AFL significantly reduced the levels of AST, ALT and MDA, improved the blood lipid levels, increased the activities of hepatic GSH-PX and SOD, and inhibited hepatocellular lesions in mice with AFL, thus exerting a certain protective effect on the hepatic tissues of mice. In conclusion, the composite modification of SG and MT could improve the physicochemical properties, microstructure and health care function of ASDF. This study provided a theoretical basis for the drug development of AFL.

## Data availability statement

The original contributions presented in the study are included in the article/supplementary material, further inquiries can be directed to the corresponding author.

## Ethics statement

The animal study was approved by the Laboratory Animal Welfare Ethics Committee of Hunan SJA Laboratory Animal Co., Ltd. (IACUC-SJA2021020; date of approval: 14 November 2021). The study was conducted in accordance with the local legislation and institutional requirements.

## Author contributions

YW: Investigation, Methodology, Project administration, Supervision, Writing – review & editing. BH: Data curation, Formal analysis, Investigation, Methodology, Writing – original draft. LZ: Methodology, Writing – review & editing. RZ: Methodology, Writing – review & editing. LH: Funding acquisition, Supervision, Writing – review & editing.

## Funding

This research was supported by LH in Hunan Province Natural Science Foundation, grant number: 2019JJ60020.

## Conflict of interest

The authors declare that the research was conducted in the absence of any commercial or financial relationships that could be construed as a potential conflict of interest.

## Publisher’s note

All claims expressed in this article are solely those of the authors and do not necessarily represent those of their affiliated organizations, or those of the publisher, the editors and the reviewers. Any product that may be evaluated in this article, or claim that may be made by its manufacturer, is not guaranteed or endorsed by the publisher.

## Glossary

**Table:**

DF	Dietary fiber
ASDF	Artichoke soluble dietary fiber
ADF	Artichoke dietary fiber
SDF	Soluble dietary fiber
MT	Microwave treatment
SG	Superfine grinding
CM	Compound modification
MT-ASDF	Microwave treatment of artichoke soluble dietary fiber
SG-ASDF	Superfine grinding treatment of artichoke soluble dietary fiber
CM-ASDF	Compound modification of artichoke soluble dietary fiber
L-ASDF	Low dose of artichoke soluble dietary fiber
M-ASDF	Middle dose of artichoke soluble dietary fiber
H-ASDF	High dose of artichoke soluble dietary fiber
BG	Blank group
CG	Control group
GSH-PX	Glutathione peroxidase
AST	Aspartate aminotransferase
ALT	Alanine aminotransferase
SOD	Superoxide dismutase
MDA	Malonaldehyde
OHC	Oil-holding capacity
WHC	Water-holding capacity
WSC	Water swelling capacity
AFL	Alcoholic fatty liver
TNF-α	Tumor necrosis factor-α
IL-6	Interleukin-6
